# Safety Aspects of Bio-Based Nanomaterials

**DOI:** 10.3390/bioengineering4040094

**Published:** 2017-12-01

**Authors:** Julia Catalán, Hannu Norppa

**Affiliations:** 1 Work Environment, Occupational Safety, Finnish Institute of Occupational Health, FI-00250 Helsinki, Finland; hannu.norppa@ttl.fi; 2 Department of Anatomy, Embryology and Genetics, University of Zaragoza, 50013 Zaragoza, Spain

**Keywords:** nanosafety, bio-based nanomaterials, toxicity testing, hazard assessment, regulations

## Abstract

Moving towards a bio-based and circular economy implies a major focus on the responsible and sustainable utilization of bio-resources. The emergence of nanotechnology has opened multiple possibilities, not only in the existing industrial sectors, but also for completely novel applications of nanoscale bio-materials, the commercial exploitation of which has only begun during the last few years. Bio-based materials are often assumed not to be toxic. However, this pre-assumption is not necessarily true. Here, we provide a short overview on health and environmental aspects associated with bio-based nanomaterials, and on the relevant regulatory requirements. We also discuss testing strategies that may be used for screening purposes at pre-commercial stages. Although the tests presently used to reveal hazards are still evolving, regarding modifi­cations required for nanomaterials, their application is needed before the upscaling or commercialization of bio-based nanomaterials, to ensure the market potential of the nanomaterials is not delayed by uncertainties about safety issues.

## 1. Introduction

The movement of our society towards a bio-based and circular economy implies a major focus on the responsible and sustainable utilization of bio-resources [1,2]. Pressure on biological resources should be reduced, and a circular economy needs to be realized by utilizing bio-waste as a resource and ensuring sustainable biomass production [3]. Renewable resources will play a key role in the production of novel bio-based materials, contributing to improving the environmental profile of polymer systems and composites currently produced from petroleum-based sources [2,4].

The forests constitute a major source of lignocellulosic biomass, a sustainable resource that, in addition, is not competing with food production [5]. Lignocellulosic materials have long been used in the paper and packing industrial sector, in healthcare products, such as wound healing materials, dialysis membranes, and excipients in tablets, and as rheology modifiers in food products and cosmetic formulations [6,7]. However, the emergence of nanotechnology has opened new and multiple possibilities, not only in the existing industrial sectors, but also for completely novel applications, such as biofuels, aerogels for insulation, vehicles’ components, electronics, or tissue engineering [2,4,6,7]. The commercial exploitation of nanoscale bio-materials has only begun during the last few years, and although it is currently at pilot-scale for many applications, large-scale commercialization is anticipated [4,6]. Therefore, companies have a unique chance of being proactive by demonstrating the safety of the emerging bio-based nanomaterials in advance of their commercialization, ensuring that their market potential is not delayed by uncertainties about safety issues [4,7].

## 2. Toxicity of Bio-Based Nanomaterials

Due to their natural origin, bio-based materials are often assumed not to be toxic. In the middle of last century, the recognition of byssinosis as an occupational lung disease, observed in workers of the textile industry exposed to cotton dust, led to several studies investigating the possible health risks associated with cellulosic materials. An excellent review of these studies has been published by Endes et al. [6]. Whereas toxicological findings were contradictory among studies, all of them showed an extremely high biopersistence of cellulose fibers. Biopersistent long and thin high-aspect-ratio fibers have been postulated to have the potential to be carcinogenic, and lead to mesothelioma and lung cancer, according to the fiber pathogenicity paradigm [8]. Nanoscale features may impart novel material properties and biological behavior, as compared with conventional materials. A decrease in particle size is associated with improved penetration through biological barriers, resulting in increased doses of materials within cellular compartments or translocation to new localizations (e.g., the brain) [9]. On the other hand, the large surface area of nanomaterials may result in enhanced interaction with their biological surroundings [10,11,12], leading in some cases to an accelerated formation of reactive oxygen species [9,12]. In light of these findings, it is necessary to address the human health and environmental safety aspects of lignocellulosic nanomaterials before scaling up their production [6,7,13,14].

Currently, little is known about the potential adverse biological impact of lignocellulose nanomaterials, which comprise a broad spectrum of different structures from cellulose nanocrystals and different types of nanofibrillated celluloses to lignin nanomaterials (see, e.g., reviews by Shatkin et al. [4], Endes et al. [6] and Roman [13]; as well as summaries of more recent articles included in Shvedova et al. [15] and Catalán et al. [16]). The scarce toxicological data are interpreted in different ways by researchers. Whereas several authors think that the available results suggest that nanocelluloses have a limited associated toxic potential [4,6,17], conflicting conclusions are reached, especially for inhalation results and cytotoxicity [13,16]. However, all authors agree on assuming an association between the hazard profile of lignocellulosic nanomaterials and their physicochemical characteristics. It is well-recognized that the physicochemical features of nanomaterials can affect their toxicity [18]. For instance, the interaction of nanofibrillated cellulose and dendritic cells depended on the thickness and length of the material [19]. Therefore, differences in production technique, which can dramatically affect the physicochemical characteristics of cellulosic nanomaterials [20], may also affect their hazard features. That is the case for surface chemistry, which has been reported to drive the inflammatory response to nanofibrillated cellulose [21]. Although most toxicological studies have focused on unmodified materials [4], for many applications, such as uses in healthcare products and food packaging, nanocellulose may be surface functionalized, imparting new properties to the material [6]. However, far from being an obstacle, the possibility of moderating biological responses by introducing surface modifications opens up the option of safe-by-design for these materials [21].

The route of exposure may also determine the toxicological responses of bio-based nanomaterials. The main portals of entry to the human body include the gastrointestinal tract, skin, systemic circulation, and the lung, through inhalation [6]. The latter is considered the primary route of exposure for humans for any nanoparticle released into the environment, especially in the case of occupational exposure [22], as also confirmed by a life cycle risk assessment of nanocelluloses [23]. In addition, nanocelluloses are expected to be biopersistent, as indicated by in vitro experiments with artificial lung airway lining and macrophage phagolysosomal fluids [24], and in vivo evidence [14,15]. As previously mentioned, biopersistence of fibers has been identified as a key factor governing the biological response following chronic inhalation exposures [8]. Therefore, the release and inhalation of cellulose/polymer particles during processing steps, such as drilling, cutting, and sanding of polymer nanocomposites [6], in addition to possible liquid aerosols in wet operations, might be a concern.

Although inhalation has been pointed out as the main route for human exposure to nanomaterials, little is known about exposure concentrations or doses [25]. Few studies have reported measurements of cellulose nanomaterials in occupational environments [17,26]. This means that there is not enough data on occupational exposure or inhalation toxicity exist for cellulose nanomaterials to determine material-specific occupational exposure limits (OELs) for airborne dusts [4]. As for other nanomaterials, lower exposure levels may be expected to be harmful for nanosized, than bulk forms. Applying the pre-cautionary principle, Stockmann-Juvala et al. [27] recommended an OEL for nanocelluloses of 0.01 fibers/cm^3^, which is the same value as suggested for other biopersistent fibrous nanomaterials, e.g., carbon nanofibers. As pointed out by these authors, the comparison of air sample concentrations with the suggested OEL is difficult at the moment, due to the lack of reliable methods for quantitative measurement. Therefore, a minimization of the exposure is recommended, as long as these methods are not available [27].

## 3. Regulatory Requirements and Testing Strategies

The identification of material-posed hazards is required under several international regulations (e.g., Registration, Evaluation, Authorization and Restriction of Chemicals—REACH, biocides, pharmaceuticals, medical devices, food additives, cosmetics, etc.) dealing with the safe use of chemical substances, products or devices (including nanomaterials-enabled ones). The corresponding regulations under which the bio-based nanomaterial will need to be evaluated in connection with scaling up of production or commercialization will depend on the uses and applications of the materials, and the countries where they apply [7]. For instance, in the European Union, approval of medical devices is addressed by three different European Commission directives [28], whereas different directives apply to pharmaceuticals [29]. In addition, commercialization of chemical substances is regulated by REACH [9,30], which aims at ensuring the protection of workers, downstream users, consumers and the environment. Furthermore, the United Nations’ Globally Harmonized System (GHS), which harmo­nizes the classification and communication of hazardous properties of chemicals worldwide [31], has been adopted in several international regulations (e.g., in Europe). In an interesting article, Shatkin et al. [4] identified the data gaps needed for cellulose nanomaterials to comply with the requirements of this system.

Although there is a broad range of regulations, most of them agree on some basic human health and environmental effects that should be addressed [7]. In general, hazard assessment relies on several endpoints (e.g., cytotoxicity, sensitization, genotoxicity, etc.) that are assessed using validated assays, most of them still requiring animal experiments. It is, however, expected that new alternative methods, in accordance with the 3 Rs principles, will substitute them in the future [32]. Therefore, the commercialization of bio-based materials will have to comply with the corresponding regulatory requirements, depending on the intended final use of the products. Nevertheless, a first screening of the toxic potential of the materials can be done at the pre-commercialization or pre-clinical stages using in vitro assays.

During the last few years, several approaches and frameworks have proposed a testing strategy suitable for nanomaterials [33,34,35,36]. Most toxicological studies on nanomaterials assess cytotoxic potential in different human or mammalian cell systems. Cytotoxicity is one of the endpoints requested for medical device biocompatibility testing to obtain regulatory approval in most markets [37]. It is also required as a pre-test for establishing the range of doses to be evaluated in more specific endpoints, such as genotoxicity. However, although cytotoxicity assays may allow a way to rank nanomaterials [38], differentiating between partly soluble and poorly soluble metal-based nanoparticles, they do not indicate which doses are toxic in vivo, or if the effect is organ-specific. Cytotoxicity neither provides information on the type of hazardous event and the possible mechanism of action. The latter two can be assessed by a battery of assays that, in addition to cytotoxicity in a relevant cell system for the route of exposure, also include more specific endpoints. Furthermore, the lack of cytotoxicity does not mean the lack of hazardous effects [39]. For instance, the woodderived nanofibrillated celluloses studied by Lopes et al. [21] did not impair the cell viability of dermal cells, lung cells, or macrophages. However, the unmodified nanofibrils promoted an inflammatory response in macrophages.

Genotoxicity is a key endpoint in the toxicity testing of nanomaterials [33,34,36,40]. It has important consequences to human health, because mutations play a crucial role in the initiation and progression of carcinogenesis, and in reproductive and developmental abnormalities [41,42]. Genotoxicity is a hazard endpoint required in all product regulations (REACH, biocides, pharmaceuticals, medical devices, food additives, cosmetics, etc.), as well as one of the categories considered within the GHS for Hazard Communication [31]. The assessment of genotoxicity is based on validated in vitro assays, which can be followed up by validated in vivo assays, depending on the in vitro outcome and the regulation involved. Therefore, genotoxicity assessment at an early stage of pre-commercialization is highly advised.

Inflammation is one of the initial steps that may give rise to lung fibrosis, secondary genotoxic effects, and carcinogenesis after inhaling biopersistent nanofibers. In addition, most of the nanomaterials administrated intravenously (e.g., nanomedicines) end up in immune-related organs containing cells of the mononuclear phagocytic system [43]. Furthermore, all new human pharmaceuticals should be evaluated for potential immunotoxic activity [44]. Therefore, immunotoxicity testing should be included in a testing strategy [34,36].

Oxidative stress has been suggested to be a mechanism often underlying the possible toxicity of nanomaterials, causing both immunotoxic and genotoxic effects [36,45]. Therefore, this endpoint is included in most of the testing strategies proposed for nanomaterials. For instance, the in vitro testing strategy suggested by Endes et al. [46] to mimic the inhalation of high aspect ratio nanoparticles includes the assessment of cytotoxicity, oxidative stress, and pro-inflammatory responses in a 3D lung model.

Bacterial lipopolysaccharides (LPS, also termed endotoxin) are common contaminators of naturally derived materials [47]. Endotoxins are known to trigger inflammation, and they may induce oxidative stress and subsequently other toxic effects (e.g., DNA damage) [16]. The absence of endotoxin in nanomaterial test samples is considered important, and should be reported in toxicity studies [48], especially in the case of immunotoxicity testing for biomedical applications [47,49]. However, endotoxin testing of nanomaterials is not straightforward, due to the interference of nanomaterials with the endotoxin assays [49]. Polymyxin B is sometimes used in parallel immunotoxicity experi­ments to inhibit the potential effects of any endotoxin present in the samples [21]. Nevertheless, producing and handling of the nanomaterials in an environment as much endotoxin-free as possible is highly advised [49]. For instance, Nordli et al. [47] have described an updated method to produce ultrapure cellulose nanofibrils suitable for wound dressings.

It is worth emphasizing that there are few legislations specifically dealing with nanomaterials (e.g., the European biocidal products and cosmetics regulations). Instead, nanomaterial safety evaluation is based on the existing regulatory frameworks and, hence, on validated standard toxicological assays. However, the ability of some of these assays to detect the potential hazards of nanomaterials has not well been established [35,43], and recommendations for assay modifications have been suggested [48]. Furthermore, an important question in toxicological testing of nanomaterials is the limited ability of the present in vitro assays to deal with secondary toxic mechanisms and organ specificity that are fully present only in a whole organism in vivo. Co-culture of e.g., inflammatory and target cells may provide a simple system for detecting secondary effects, such as secondary genotoxicity [40], although such approaches have not widely been evaluated. 3D tissue models have received increased attention in toxicology, but only a limited number of studies utilizing these techniques has thus far been carried out with nanomaterials [50]. The integration of new technologies (e.g., “omics”) may in the future allow researchers to elucidate mechanistic pathways involved in toxicological responses of nanomaterials [40]. In the meantime, assay limitations should be taken into account when using conventional in vitro tests for the hazard identifications of bio-based nanomaterials.

## 4. Environmental Issues

Finally, effects on the environment are also part of safety evaluation. Bio-based materials are assumed to be environmentally friendly [51,52]. In fact, wood-based feedstocks have much shorter carbon cycle and lower greenhouse gas emissions than fossil-based feedstocks [2]. In addition, the use of wood-based materials can represent a bio-based alternative that does not compete with food production, since forest areas are usually unsuitable for food production [2]. However, the ecotoxicological data available by now are not adequate to allow the environmental hazard classification of cellulose nanomaterials [4] or bio-based materials in general. Many bio-based materials are regarded as readily biodegradable [4], but this is not true for all of them. For instance, some plastics are not really biodegraded, but they break down into very small fragments that accumulate in the environment [2]. Even for completely biodegradable materials, there are limited data regarding their bioaccumulative potential and soil mobility [4], which prevents the exclusion of environmental concerns.

## 5. Conclusions

Although legislation specifically concerning the safety of nanomaterials is still rare, existing regulations governing the risks of chemical substances, biocides, pharmaceuticals, medical devices, food additives, cosmetics, etc. are in effect, and also apply to bio-based nanomaterials. Despite the tests presently used to reveal hazards to human health and the environment are still evolving, regarding modifications required for nanomaterials, their application is needed before the upscaling or commercialization of bio-based nanomaterials.
